# Relation Between Increased Fetal Nuchal Translucency Thickness and Chromosomal Defects in Northern Vietnam

**DOI:** 10.7759/cureus.18446

**Published:** 2021-10-02

**Authors:** Nguyen Hai Long, Tran Danh Cuong, Ngo Toan Anh

**Affiliations:** 1 Department of Obstetrics, Haiphong Hospital of Obstetrics and Gynecology, Haiphong, VNM; 2 Department of Obstetrics and Gynecology, Hanoi Medical University, Hanoi, VNM; 3 Department of Obstetrics and Gynecology, National Hospital of Obstetrics and Gynecology, Hanoi, VNM

**Keywords:** amniotic fluid, amniocentesis, chromosome aberrations, vietnam, karyotype analysis

## Abstract

Objective

To examine the prevalence of all chromosomal defects amongst fetuses with increased nuchal translucency thickness (NT).

Methods

This is a retrospective study amongst pregnant women indicated for amniocentesis by nuchal translucency above 3.0 mm and consent to the study. A total of 2,720 cases were recruited during the six-year period from 2015 to 2020. All singleton pregnancies were offered fetal karyotype when the fetal nuchal translucency was ≥2.5 mm. The prevalence of chromosomal defects was divided into five NT categories: 2.5-3.4 mm, 3.5-4.4 mm, 4.5-5.4 mm, 5.5-6.4 mm, ≥6.5 mm.

Results

The study identified 2,720 amniocentesis for increased NT. The mean maternal age was 29.19 (range 17-46) years, and the mean fetal crown-rump length was 66.9 (range 45-84) mm. The fetal karyotype was abnormal in 560 (20.6%) participants. The most frequent chromosomal disorders were trisomy 21 (55%), trisomy 18 (11.2%), trisomy 13 (3.9%), 45,XO (2.7%). The prevalence of chromosomal aberrations was ranged from 17.9% (NT between 2.5-3.4 mm) to 29.7% (NT≥6.5 mm). A majority of fetuses with trisomy 13, 18, or 21 has NT measurement lower than 5.5 mm. In those with Turner syndrome, there was no difference between the group with NT <5.5 mm and the group with NT ≥5.5 mm. Increased maternal age is a risk factor for chromosomal aberrations with the rate increased from 17.6% at the youngest maternal age of 30-34 to 34% at maternal age of 35-39 and to 50% at maternal age of ≥40.

Conclusion

In fetuses with increased NT, more than a half of the chromosomal abnormalities were affected by defects other than trisomy 21. The distribution of NT was different between Turner syndrome and trisomy 13, 18, 21 syndromes. Women aged 35 years or older had a higher risk of chromosomal aberrations.

## Introduction

Fetal nuchal translucency (NT), in the first trimester of pregnancy, is an ultrasonographic image of a subcutaneous assembly of fluid behind the fetal neck, and this terminology is used irrespective of whether the fluid is septated or not and whether it is found only at the fetal neck or covered the whole fetus [1]. Nuchal translucency is defined as increased if the vertical thickness, measured in the midsagittal section of the fetus, is equal to or greater than 3.0 mm during pregnant screening [2]. Amniocentesis is one of the most popular invasive procedures for prenatal diagnosis that have begun decades ago, which was initially performed to drain fluid in the treatment of polyhydramnios. After a year of the successful establishment of the fetal chromosome map by amniotic fluid culture, the structural re-arrangement of the fetal chromosome was approved in 1967 [1]. In 1968, Valenti and colleagues diagnosed aneuploidy of trisomy 21 via amniotic fluid culture [3]. Since then, the method of obtaining fetal specimens by amniocentesis in prenatal diagnosis has been globally applied [4-6].

Increased NT is associated with trisomy 21 (T21) and other chromosomal aberrations as well as fetal structural defects and genetic syndromes [7-9]. Previous studies highlight that increased NT, both founded alone and in combination with other ultrasound findings or maternal serum biochemical markers, could be a significant indicator for first-trimester screening for trisomy 21 [7]. This study was designed to identify the prevalence of all chromosomal defects and examine their distribution in fetuses with increased NT [10].

## Materials and methods

Methodology

This study is a clinic-based retrospective study. Pregnant women with increased NT were recruited from 19 satellite hospitals in Northern Vietnam. NT was assessed by amniocentesis performed at the Center for Prenatal Diagnosis, National Hospital of Obstetrics and Gynecology in Hanoi, Vietnam. We collected information during two periods. Data based on medical records which were archived in the Center for Prenatal Diagnosis was collected for the first three-year period (2015 to 2018), and data were collected directly from patients in the Center for prenatal diagnosis for the second two-year period (2019 to 2020). All pregnant women with gestational age ranged 11-14 weeks were scanned. The maternal characteristics and ultrasound findings, NT, and CRL in millimeters were entered and stored using an e-database. Karyotype results were added right after the available results.

Variables, data analysis

Cytogenetic findings were analyzed and classified into three categories: (1) aneuploidy of autosomal chromosomes, (2) sex chromosome aneuploidy, (3) structural re-arrangements. Patients were consulted about their clinical diagnosis by both obstetricians and geneticists before coming to their final decision of the pregnant outcomes. Pregnant-related complications such as miscarriage, bleeding, amniotic infection were noted as well as the failure to harvest adequate cells after culturing. After receiving amniotic fluid, a standard G-banding technique was applied to analyze their karyotypes. Our data were collected, entered, and analyzed using SPSS 20.0 (IBM Corp., Armonk, NY). Categorical statistics were summarized in frequency distribution tables. The prevalence and allocation of chromosomal defects were estimated by the four NT categories: 2.5-3.4 mm, 3.5-4.4 mm, 4.5-5.4 mm, 5.5-6.4 mm, ≥6.5 mm.

Ethical approval

This study was approved by the Ethical Committee of National Hospital of Obstetrics and Gynecology with the number of IRB: 457/CN-PSTW. Participants were introduced, informed of the study objectives, and participated (written consent form). Participants could withdraw when they wanted at any time, and their information was kept confidential.

## Results

Over a five-year period (2015 to 2020), among the total of 18,499 pregnant women undergoing amniocentesis at the Center for Prenatal Diagnosis, National Hospital of Obstetrics and Gynecology in Vietnam, 2,720 cases (14.7%) had increased NT (Table 1). The data presented a speeding up of the rate from 15% in 2015 to 18.2% in 2019. The prevalence of chromosomal aberrations, on average, is 20.6%. Between 2015 and 2020, the rate increased from 17.7% to 25.6%.

**Table 1:** The proportion of increased NT among number of amniocentesis each year and the rate of chromosomal aberrations. NT: nuchal translucency.

Year	Number of amniocentesis	Percentage of amniocentesis (%)	Number of NT increased	Percentage of NT increased (%)	Number of chromosomal aberrations	Percentage of chromosomal aberrations (%)
2015	3192	17.3	480	15.0	85	17.7
2016	2903	15.7	129	4.4	25	19.4
2017	3091	16.7	476	15.4	90	18.9
2018	3182	17.2	550	17.3	117	21.3
2019	3301	17.8	601	18.2	119	19.8
2020	2830	15.3	484	17.1	124	25.6
Total	18499	100	2720	14.7	560	20.6

Table 2 presents the maternal age-specific distribution of women with increased NT. Regarding the age group of the pregnant women with increased NT, most of them aged 20-35 years old, younger women (19 years old or lower) accounted for only 1.6%, and older women (35 years old or higher) accounted for 16.3%. The prevalence of chromosomal aberrations increased with ages: about 18% amongst women aged 34 or lower and nearly doubled amongst women aged 35-39 years old (34%) and tripled amongst women aged 40 years old or higher (50%).

**Table 2:** Maternal age-specific distribution of women with increased nuchal translucency thickness.

Maternal age	Number	Percentage (%)	Number of chromosomal aberrations	Percentage of chromosomal aberrations (%)
≤ 19	43	1.6	8	18.6
20-24	441	16.2	79	17.9
25-29	1042	38.3	172	16.5
30-34	752	27.6	132	17.6
35-39	326	12	111	34
≥ 40	116	4.3	58	50
Total	2720	100	560	20.6

Table 3 presents the incidence of Chromosomal Defects in fetuses with increased NT. The most common type of chromosomal aberrations was trisomy 21 (53.9%), followed by trisomy 18 (12.1%), and trisomy 13 (3.4%). Regarding sex chromosomal abnormalities (6.2%), 45, XO (2.3%) accounted for the highest rate. The majority of structural re-arrangements were inversion (2.9%), polymorphism (10.4%), Robertsonian translocation (2.7%).

**Table 3:** Incidence of chromosomal defects in fetuses with increased nuchal translucency thickness.

Chromosomal aberrations	Case number	Proportion (%)
1. Autosomal aneuploidy	399	71.3
Triploidy	1	0.2
Trisomy 21	302	53.9
Trisomy 18	68	12.1
Trisomy 13	19	3.4
Mosaicism	2	0.4
Other chromosomes	7	1.3
2. Sex aneuploidies	35	6.2
45, X	13	2.3
47, XXY	3	0.5
47, XXX	7	1.3
47, XYY	4	0.7
Mosaicism	8	1.4
3. Structural rearrangements	125	22.5
Reciprocal translocation	9	1.6
Robertsonian translocation	15	2.7
Der translocation	5	0.9
Inversion	16	2.9
Deletion	5	0.9
Duplication	0	0.0
Insertion	4	0.7
Marker chromosomes	2	0.4
Polymorphism	58	10.4
Mosaicism	11	2.0
Total	560	100

Fetal karyotype was performed in all 2720 singleton pregnancies with high NT. The incidence of chromosomal aberrations is presented in Table 4. The mean maternal age was 29.19 years old (range 17-46), and the mean fetal CRL was 66.9 mm (range 45-84). About 560 (20.6%) pregnancies had abnormal fetal karyotype, whereas women with trisomy 21 were the highest group (Table 4). The overall prevalence of chromosomal defects increased with more increased NT from 17.9% for those with NT of 2.5-3.4 mm to 21.2% for those with NT of 3.5-4.4 mm, 26.6% for those with NT of 5.5-6.4 mm, and 29.7% for those with NT of 6.5 mm or higher. Regarding the fetuses with trisomy 21, with trisomy 13 or 18, most of the cases had the NT <5.4 mm (90.9%, 90.9%, and 87.3%, respectively) while only a half of patients with Turner syndrome (53.3%) had NT ≥5.5 mm.

**Table 4:** Classifications of chromosomal aberrations following nuchal translucency thickness.

Nuchal translucency (mm)	Mean maternal age [range (y)]	N	Abnormal karyotype							
			Total	Trisomy 21	Trisomy 18	Trisomy 13	Turner syndrome	Sex	Triploidy	Other
2.5-3.4	29.23 (17-46)	1372 (50.4)	245 (17.9)	122 (49.8)	27 (11.0)	9 (3.7)	5 (2.0)	5 (2.4)	0	76 (31)
3.5-4.4	29.36 (18-45)	866 (31.8)	182 (21)	114 (62.6)	15 (8.2)	9 (4.9)	2 (1.1)	6 (3.3)	1 (0.5)	35 (19.2)
4.5-5.4	29 (18-45)	282 (10.4)	77 (27.3)	44 (57.1)	13 (16.9)	2 (2.6)	0	1 (1.3)	0	17 (22.1)
5.5-6.4	28.39 (17-42)	109 (4.0)	29 (26.6)	12 (41.4)	6 (20.7)	1 (3.4)	4 (13.8)	0	0	6 (20.7)
≥ 6.5	28.46 (18-42)	91 (3.3)	27 (29.7)	16 (59.3)	2 (7.4)	1 (3.7)	4 (14.8)	0	0	4 (14.8)
Total	29.19 (17-46)	2720	560 (20.6)	308 (55)	63 (11.2)	22 (3.9)	15 (2.7)	13 (2.3)	1 (0.2)	138 (24.7)

## Discussion

This study is one of the first retrospective study (over a five-year period) with a high sample of women performing amniocentesis conducted in Northern Vietnam. The most frequent chromosomal disorders were trisomy 21 (55%), trisomy 18 (11.2%), trisomy 13 (3.9%), 45, XO (2.7%). Amniocentesis is an invasive procedure for collecting specimens directly from the fetus to evaluate fetal genetic conditions. For abnormal cases, fetal karyotype was performed to support the final decision for pregnant women. Performing fetal karyotype for abnormal cases is a regular process not only in Vietnam [11] but also in Austria (82.44%) [12].

A total of 2720 pregnant women with high NT, which accounted for 14.7% of all cases performing amniocentesis, were found after the routine ultrasonography for all pregnancies during the gestational age of 11-13 + 6 weeks. The incidence of chromosomal abnormality, which was highest in the last year of the study (2020) with 25.6%, is in line with other studies worldwide [5,6,13]. The result should be explained that the Center for Prenatal Diagnosis at Vietnam, National hospital of Obstetrics and Gynecology, is the specialized and highest professional Center in Northern Vietnam for diagnosing high-risk pregnancies. The incidence of chromosomal aberrations, therefore, could be a representative number for high-risk fetuses in Northern Vietnam. Notably, our results proposed that increased NT is one of the most common criteria for high-risk fetuses (20.6% versus 6.7% of other causes). This suggested that the government should widely perform the low-cost and effective test for NT to all high-risk pregnant women. It is a very effective method for aneuploidy screening in the first trimester.

Maternal age in combination with increased NT was significantly associated with chromosomal aneuploidy. In our study, the incidence of chromosomal aberrations risen rapidly from 17.6% amongst women aged 30-34 years old to 34% and 50% amongst women aged 35-39 years old and aged ≥40 years old, respectively. This is in line with previous studies reporting that pregnant women aged 35 years old and older with high NT are more likely to have abnormal karyotype [5,6,13].

In our study, the incidence of fetuses with trisomy 21 had the highest proportion with 302 cases (53.9%), followed by trisomy 18 (12.1%), polymorphism (10.4%), and trisomy 13 (3.4%). Other chromosomal defects had the incidence rate of about 3% or lower. Similar to our results, chromosomal structure abnormalities are rare in prenatal diagnosis [14,15]. Data in previous studies have shown that the incidence of visible structure abnormalities was about 23.7% [11], a bit higher than that of our study (22.5%). High NT not only increases the risk of chromosomal aneuploidy but also of structural re-arrangements. In Vietnam, abortion is legally approved if detecting any prenatal genetic disorders. In our study, most pregnant women terminated their pregnancies after having the result of karyotype.

Similar to our results, a significant association between increased NT and trisomy 21 as well as other chromosomal defects like trisomy 13, 18 and sex aneuploidy were previously reported [7,8,16]. Our results found that trisomy 21 was found in nearly half of the abnormal chromosomal fetuses. Furthermore, the distribution of NT was different for each type of chromosomal defect. The NT <5.5 mm appeared in 90.9% of fetuses with trisomy 21, trisomy 13, and trisomy 18, while the NT ≥5.5 mm appeared in 53.3% of fetuses with Turner syndrome. NT in other sex abnormalities was mostly <5.5 mm.

Mechanisms for increased NT differ between chromosomal aberrations. For trisomy 18, it includes cardiac function failures in association with structural defects of the fetal heart and great arteries [17,18]; superior mediastinal compression due to diaphragmatic hernia [19,20]. For Turner syndrome, it’s the impaired lymphatic drainage due to damaged development of the lymphatic system. Literately, immunohistochemical studies have been demonstrated in nuchal skin tissue [21]; and subcutaneous connective tissue structure modifications, leading to the accumulation of subcutaneous edema [22,23] collagen. Although cardiac impairments are popularly recorded with all major chromosomal abnormalities, there were variations in the types of cardiac defects, resulting in different severities of cardiac dysfunction [17,18]. The extracellular matrix protein components are encoded on chromosomes 21, 18, or 13. Several immunohistochemical studies of the skin of chromosomally abnormal fetuses have shown certain malformations of the extracellular matrix that may be attributed to gene expression effects. Therefore, the dermis of trisomy 21 fetuses is rich in collagen type VI. In contrast, dermal fibroblasts of trisomy 13 fetuses demonstrate an abundance of collagen type IV and trisomy 18 fetuses an abundance of laminin [22,23].

Strengths and limitations

We have a large number of amniocenteses and a wide range of chromosomal defects. This study could bring us the situation of chromosomal defects in increased fetal nuchal translucency in Vietnam. More comprehensive studies are needed to screen infants who were born with structure re-arrangement. In this research, we still couldn’t have the CMA and WES in our Center. But for further study, we would like to indicate CMA and WES to the protocol to identify the CNV in order to clarify more structural disorders to complete the results of the invasive test and to strengthen the prognosis of the infant in the post-natal period.

## Conclusions

We suggested that in routine prenatal screening for pregnant women in their first trimester, increased NT was an effective marker not only to detect trisomy 21 but also to other major chromosomal defects, including trisomy 13, 18, Turner syndrome, sex abnormalities, and chromosomal structural re-arrangements especially amongst women aged 35 years old and older.
